# A spiking neural network model of the Superior Colliculus that is robust to changes in the spatial–temporal input

**DOI:** 10.1038/s41598-022-10991-6

**Published:** 2022-04-28

**Authors:** Arezoo Alizadeh, A. John Van Opstal

**Affiliations:** Dept. Biophysics, Donders Centre for Neuroscience, Radboud University, Heyendaalseweg 135, 6525 EZ Nijmegen, The Netherlands

**Keywords:** Biophysics, Computational biology and bioinformatics, Neuroscience

## Abstract

Previous studies have indicated that the location of a large neural population in the Superior Colliculus (SC) motor map specifies the amplitude and direction of the saccadic eye-movement vector, while the saccade trajectory and velocity profile are encoded by the population firing rates. We recently proposed a simple spiking neural network model of the SC motor map, based on linear summation of individual spike effects of each recruited neuron, which accounts for many of the observed properties of SC cells in relation to the ensuing eye movement. However, in the model, the cortical input was kept invariant across different saccades. Electrical microstimulation and reversible lesion studies have demonstrated that the saccade properties are quite robust against large changes in supra-threshold SC activation, but that saccade amplitude and peak eye-velocity systematically decrease at low input strengths. These features were not accounted for by the linear spike-vector summation model. Here we show that the model’s input projection strengths and intra-collicular lateral connections can be tuned to generate saccades and neural spiking patterns that closely follow the experimental results.

## Introduction

### Background

Saccades are fast eye movements that redirect the fovea to a peripheral target. They obey a stereotyped ‘main-sequence’ kinematic relationship between saccade amplitude and movement duration (an affine relation) and between amplitude and peak eye-velocity (a saturating function^1^). As the duration of the acceleration phase is roughly constant across a wide range of amplitudes, saccade-velocity profiles are positively skewed, whereby skewness increases with saccade duration^2^. Moreover, because trajectories are nearly straight, the horizontal and vertical saccade-velocity profiles are scaled versions of each other, thereby approximately matched in duration and shape^3–6^.

Together, these kinematic features betray nonlinear processing in the generation of saccades. In earlier models of saccade control, the saturation of peak eye velocity was believed to reside in the (passive) saturation of firing rates of brainstem pre-motor burst neurons^7,8^. Later studies, however, have suggested that these properties may instead betray a deliberate optimal control strategy that aims to optimize speed-accuracy trade-off in the presence of multiplicative and additive neural noise^9–13^. Single-unit recordings and quantitative modelling of instantaneous spiking behavior of saccade-related cells in the midbrain Superior Colliculus (SC) have suggested that such a mechanism might be implemented at this oculomotor midbrain level^13–15^.

The SC is a primary source of gaze-motor commands to the brainstem saccade generators^8,14,16–21^, and is recruited for all voluntary and involuntary saccades. Its deeper layers contain an eye-centered topographic map of visuomotor space^16,19,22^ , in which the location and total spike count of the neural population encode the saccade amplitude and direction^17–19^. Several studies have suggested that the temporal firing profiles of the neural population may also specify the instantaneous saccade trajectory and its velocity profile^13,14,23–26^.

Although also the frontal eye fields (FEF) and posterior parietal cortex (PPC) are strongly involved in saccades, their major role appears to be in the preparation of higher-level, reward-contingent, and task-relevant eye-movements, like anti-saccades^27^, target selection and identification^28^, saccade suppression^29^ (also when its planning is already in progress^30^), or towards remembered targets^31,32^. Their main outputs are transferred to the SC, which thus constitutes a final common pathway for saccade initiation and control.

Schiller and colleagues examined the effects of FEF and SC ablations on eye movements^33^. The deficits caused by a lesion of either structure appeared to be rather subtle when monkeys were tested a few days later and recovered over time. However, when both structures were removed, monkeys were no longer able to redirect their gaze to peripheral targets. In contrast, Hepp et al.^34^ reported a strong reduction (near-abolition) in frequency and velocity of visual-evoked spontaneous saccades and quick phases of vestibular nystagmus immediately following bilateral muscimol-induced SC inactivation, indicating a crucial role for the SC output to voluntary and involuntary saccades. Also, acute FEF inactivation strongly affects the properties of visual-evoked saccades^31,32^. Thus, the immediate effects of SC and FEF inactivation seem to be much stronger than seen with the earlier longer-term ablation studies^28,33^. Presumably, the FEF can take over SC function during the recovery period, when the latter is no longer available.

Recently, Peel and colleagues examined the acute influence of inactivating FEF by local cooling on saccade metrics and kinematics and on the associated neural firing patterns of saccade-related SC cells for different saccade tasks^35^. Their results indicated that FEF inactivation did not significantly affect direct visual-evoked saccades but led to a significant decrease of about 10% in SC spiking activity for memory-guided saccades. The authors suggested that these cortically mediated saccades may utilize, besides the direct FEF-SC-brainstem pathway, an additional, flexible processing circuit that bypasses the SC.

### Problem statement

In the present paper we focused on the encoding of saccades, generated by the direct cortical-SC-brainstem pathway. Single-unit recordings of saccade-related cells in the SC have indicated that the peak firing rate, burst duration, and shape of the burst profile of the central neuron in the population depend systematically on its location in the map according to a monotonic rostral-caudal gradient^14^. Moreover, each SC neuron elicits about a fixed number of spikes for its preferred saccade, irrespective of its motor map location.

In our earlier work^14,15,41^, these features were incorporated in a simple neuro-computational feedforward spiking neural network model, in which each spike of each recruited neuron encodes a fixed (tiny) movement contribution to the saccade that is solely determined by its location (the cell’s ‘spike vector’). The saccade trajectory then results from dynamic linear summation of all spike vectors from the spike trains from all cells in the population. Because linear spike-vector summation, in combination with a linear brainstem model could reproduce the full repertoire of (nonlinear) saccade kinematics and their trajectories, we argued that the firing patterns within the SC motor map were responsible for the nonlinear main-sequence properties, velocity profiles, and component cross-coupling of saccades^14,15^. The SC motor map would thus embed an optimal control for saccade generation^13^.

**Figure 1:**
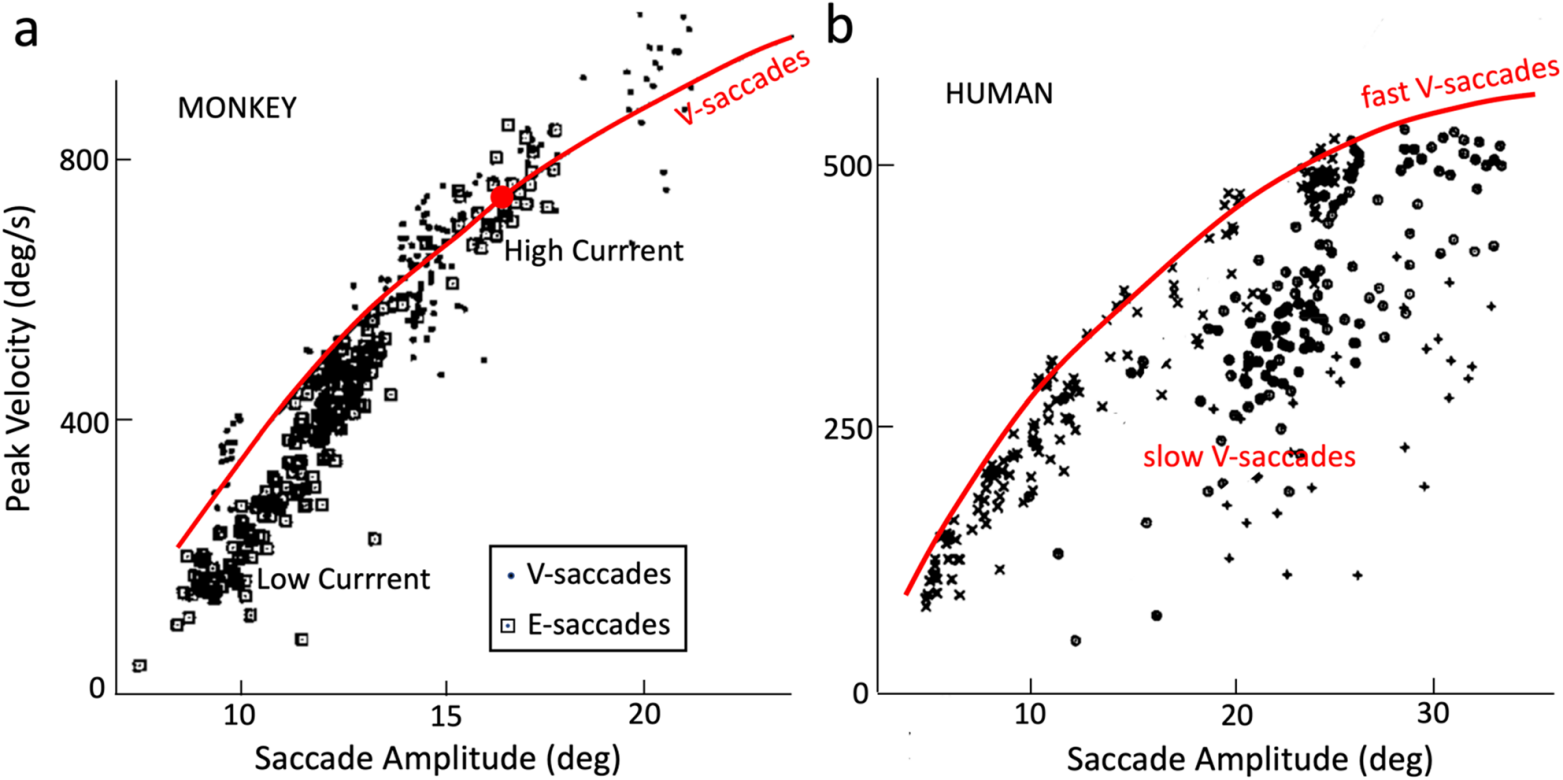
(**a**) Main-sequence relation of peak eye-velocity vs. saccade amplitude of monkey saccades for visually evoked (V-)saccades (dots) and for saccades elicited by electrical microstimulation at a single site in the superior colliculus at various current strengths (E-saccades; squares). The stimulation site in the motor map corresponded to a saccade amplitude of about 15 deg. For high stimulation currents, E-saccades and V-saccades had the same kinematics (large red dot). For the low stimulation currents at the site, two effects were observed: (i) evoked saccade amplitudes decreased, and (ii) evoked peak velocities decreased too, but fell systematically below the V-saccade main-sequence relation. Data adapted from^36^. (**b**) Human main-sequence relation for fast V-saccades (data points near the red curve) and for slow V-saccades, due to an intraveneous injection of diazepam, and fatigue. Data adapted from^2^.

Electrical microstimulation in the SC has revealed that the evoked E-saccade amplitude varies systematically with the applied current strength: at low currents, amplitudes are small, increasing to a site-specific maximum at higher current strengths, determined by the electrode’s position in the motor map^36–38^. In addition, small saccades evoked at low intensities are also slower than visual-evoked main-sequence saccades (V-saccades) of the same amplitude (Fig. 1a). Further, variation of the stimulation pulse rate affects the eye velocity: high pulse rates lead to higher saccade velocities than low pulse rates (^37^, in monkey;^39^, in barn owl). The main-sequence properties of fast and slow human V-saccades appear to follow similar kinematic characteristics^2^ (Fig. 1b). So far, these input-dependent properties had not been accounted for by our earlier linear ensemble-coding model^40^.

### This study

Here, we extended the spiking neural-network model of^40,41^ with the aim to yield similar behaviors as illustrated in Fig. 1: an increased robustness of the SC output to large variations in spiking input patterns above a certain input strength, and a systematic decrease of saccade amplitude and kinematics at lower inputs^36–38^. To simplify the analysis, we constructed a one-dimensional model with a cortical input layer and a collicular output layer and re-tuned the intra-collicular excitatory-inhibitory synapses and top-down connections. We independently varied the input spiking patterns in the spatial (i.e., population extent) and temporal (burst durations and peak firing rate) domain, reminiscent to the presumed effects of electrical stimulation, partial inactivation, or visual stimulation at different intensities, and input stimulus durations.

## Methods

### Network architecture

**Figure 2:**
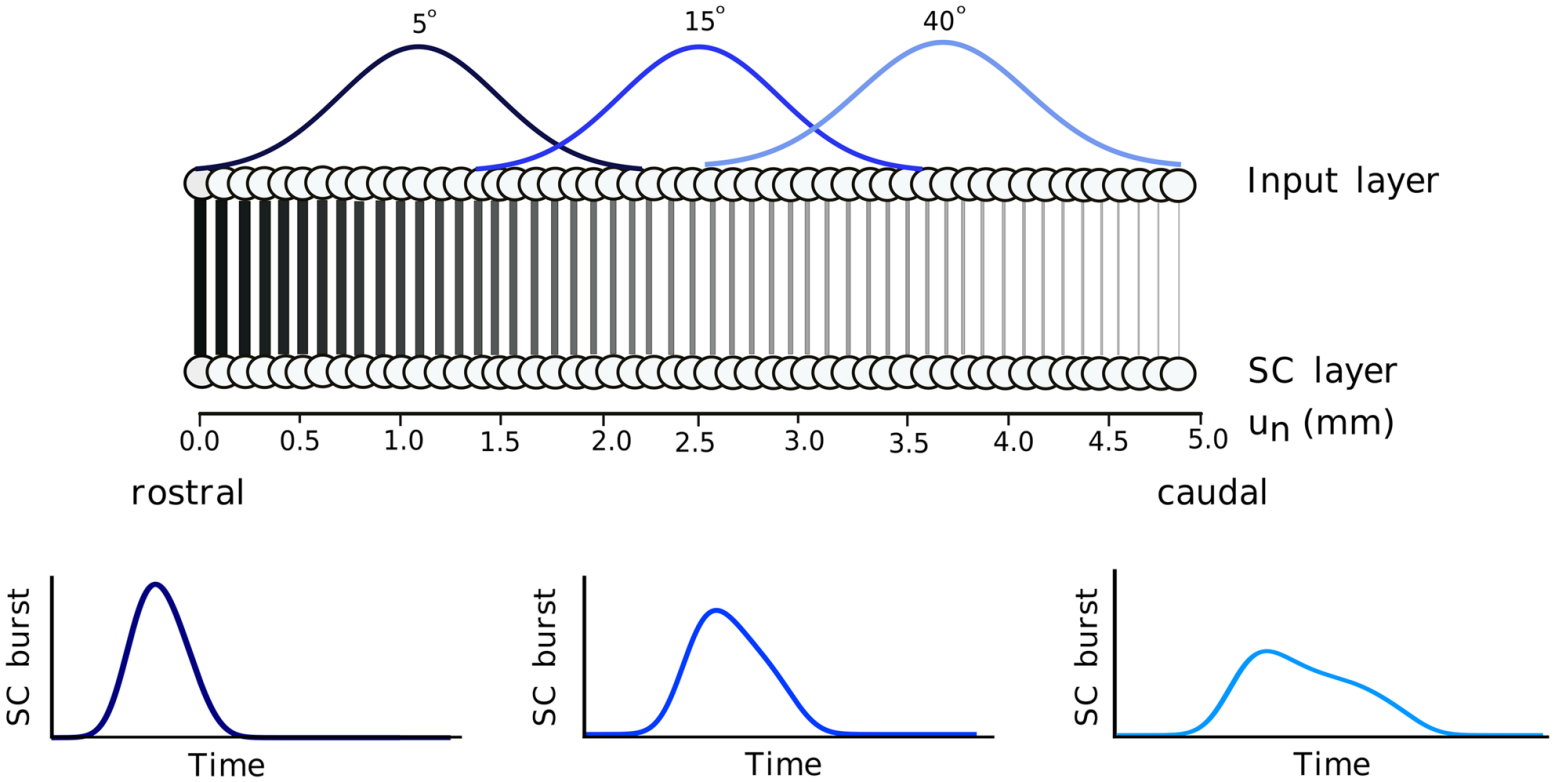
Schematic overview of the two-layer feedforward neural network. The spiking neural network model generates different saccade-related bursts (bottom) that are evoked by a spatially translation-invariant input population (top), here positioned at T = 5, 15 and 40 deg eccentricity. Thickness of the lines for the downward projections symbolizes the synaptic connection strengths, ${w}_{n}^{FS}$, between the input and SC layers (high for the rostral zone, low for the caudal zone; Eq. (3)).

We constructed a two-layer spiking neural network model with a cortical input layer, and a layer of SC output neurons, respectively (Fig. 2). Each layer consists of 200 neurons, uniformly distributed on 0–5 mm of the horizontal meridian of the SC motor map. In the linear dynamic ensemble-coding model^13,14^, the saccade kinematics are fully determined by dynamic cumulative summation of all spike vectors in the neural population during the saccade (see Supporting Information). The input layer receives an external input signal from other (here unspecified) inputs, which it transforms into spiking activity through its neural dynamics. All neurons in the model are governed by the adaptive exponential integrate-and-fire (AdEx) neural model equations (see Supporting Information, for further details; also see Ref.^41^). For simplicity, the input-layer neurons do not interact with each other. The input-layer spikes are subsequently transmitted by topography-preserving one-to-one synaptic connections to the neurons in the SC layer. The biophysical parameters of the SC neurons, such as their adaptation time constant, their synaptic connection strengths with the input layer, and their lateral excitatory-inhibitory connections, are assumed to depend on their location in the motor map and, as a result, identical firing rates in the input layer at different locations will lead to dissimilar responses of the SC cells (Fig. 2, bottom). As described below, the network is tuned such that these responses, and the ensuing saccade (equation (S1), in Supporting Information) follow similar characteristics as observed in the electrophysiological recordings and microstimulation experiments^13,36–38^.

The one-dimensional model was simulated with the Brian2 spiking neural network simulator^42^. We modeled the neurons in the network by the AdEx neuron model^43^, as the parameters of this model can be readily related to physiological quantities. Details of this neural model, including the chosen parameter values, are provided in the Supporting Information, S1 Table, and Ref.^41^. Here, we only highlight the major differences with the earlier model.

### External input current

We provided an external input current to the network around the image point, *u*_*T*_, of the desired target, *T*, in the input layer, leading to an input population spiking activity centered around the image point, *u*_*T*_ (equation (S3); Fig. 2, top). The central neuron in the input population receives the maximum input activation current, *I*_*0*_*(t)*, while the other neurons in the input layer are stimulated by current strengths that decay as a Gaussian with distance from *u*_*T*_. The spatial–temporal external input current was thus described by a separable spatial–temporal function on the input neurons by:

1 $${I}_{ext}\left({u}_{n} , t\right)={I}_{0} {\text{exp}}\left(-\frac{{\left|\left|{u}_{n} - {u}_{T}\right|\right|}^{2}}{2{\sigma }_{pop}^{2}}\right)\cdot {t}^{\gamma }\mathrm{exp}\left(-\beta t\right)$$

where *u*_*n*_ is the anatomical position of a neuron in the input map, *σ*_*pop*_ determines the size of the recruited input population, *t* is time (in s), *u*_*n*_ is the location of neuron *n* (mm), and *I*_*0*_ is the maximum input amplitude (pA). The time-dependent term is a gamma function, characterized by γ (skewness, dimensionless) and β (measure for inverse duration, in s^−1^).

To investigate the relationship between the resulting saccade metrics, trajectories, and kinematics as function of the input current profiles, we varied the input current in both the spatial and the temporal domain. The default input stimulation profile (serving as the model’s control condition) was defined by the following parameters: I_0_ = 3.0 pA, σ_pop_ = 0.5 mm, β = 0.03 s^−1^, and $\gamma$ = 1.8.

#### Spatial input variation

**Figure 3:**
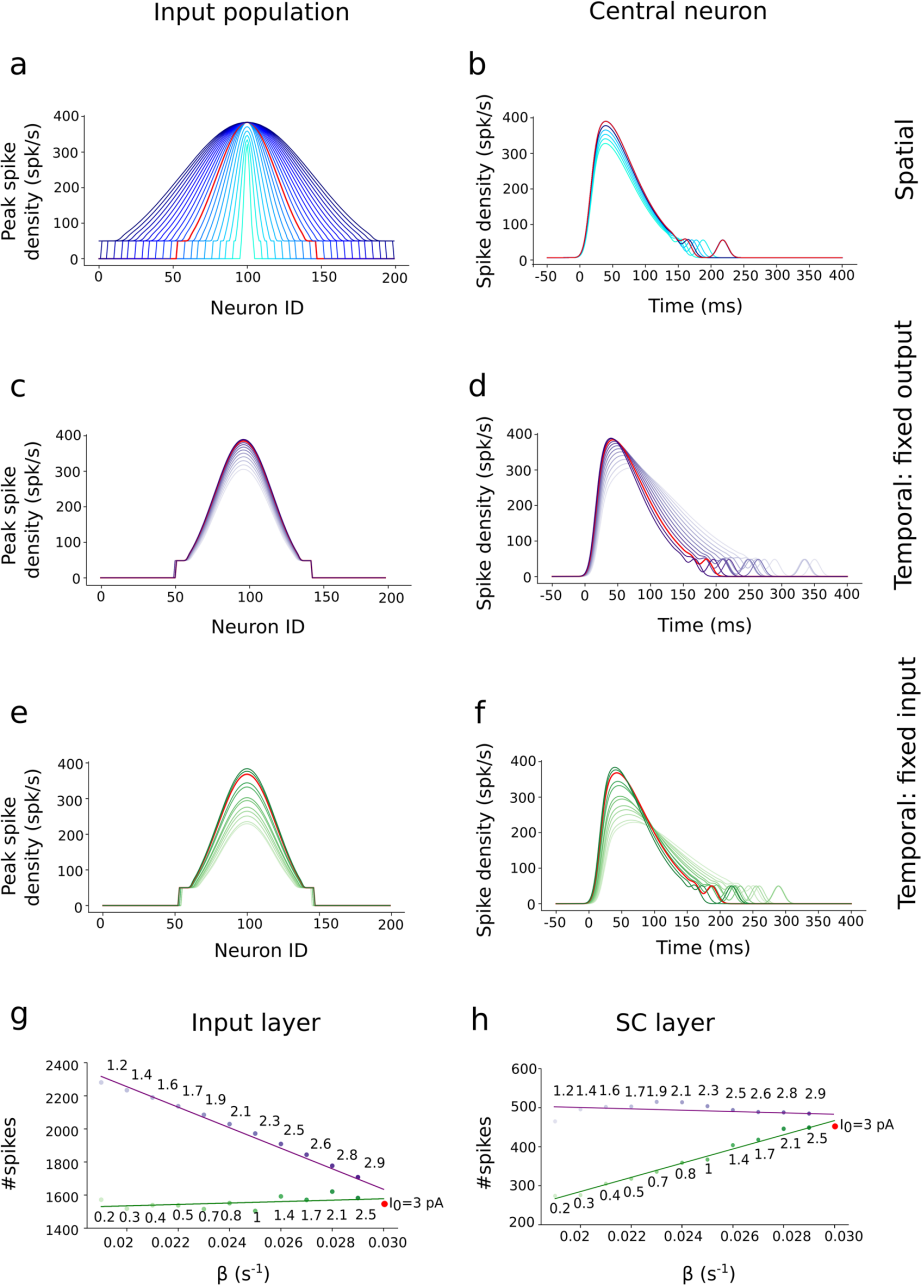
(**a**–**f**) Burst profiles of the input layer neurons in response to changes in the spatial–temporal parameters of the external input currents (see also S1 Table in Supporting Information). Red curve corresponds to the default parameter set. (**a**) Spatial distribution of the peak spike-density functions for σ_pop_ in 0.05–1.0 mm, and I_0_ in 2.0–3.0 pA at T = 15 deg. (**b**) Spike densities as function of time for the central neuron of the populations in (**a**). (**c**,**d**) Spatial (**c**) and temporal (**d**) spike-density distributions for β in 0.019–0.03 s^−1^, and I_0_ in 1.2–3.0 pA at the T = 15 deg site (σ_pop_ = 0.5 mm_)_. Parameter values of the input currents were chosen such that the SC output generated a fixed number of spikes. (**e**,**f**) Spatial (**e**) and temporal (**f**) spike-density distributions for β in 0.019–0.03 s^−1^ and I_0_ in 0.2–3.0 pA. Parameters now ensured a fixed number of spikes in the input layer. (**g**) Total number of spikes in the input layer for the two temporal variation scenarios (green and purple data). (**h**) Same as in (**g**) for the output layer.

In the spatial simulations, we varied the stimulated input population size between σ_pop_ = 0.05–1.0 mm. Input amplitudes varied between I_0_ = 2.0–3.0 pA for input population sizes below 0.5 mm and it was kept constant at 3.0 pA for input population sizes exceeding 0.5 mm. The temporal stimulation parameters were kept fixed at their default values: β = 0.03 s^−1^ and $\gamma$ = 1.8. This parameter variation led to the activation of 10–200 input-layer neurons (e.g., Fig. 3a,b).

#### Temporal input variation

To investigate the influence of input spike rates (firing frequency) to the SC motor output and SC population activity, we also varied the input current in the temporal domain. In these simulations, the externally applied input current always activated a fixed population size of σ_pop_ = 0.5 mm.

It should be noted that once the network is tuned to a particular default input duration (here, 150 ms), exceeding this duration with the current amplitude fixed at I_0_ = 3 pA will generate saccade amplitudes that will exceed the target site-specific value (even though the SC population size remains invariant, as it is normalized by the lateral interactions). The reason for this is that our model does not incorporate an additional offset mechanism that would trigger the brainstem omnipause neuronal gating system, which should prevent accessibility of SC output to the saccadic burst generator, once a fixed number of SC spikes (here, about 500 spikes, see Fig. 3h) is reached (see, e.g.^14^ for an implementation of this idea, and the Discussion section). In our simplified model (Eq. S1), every SC spike counts and therefore contributes a (small) spike-vector to the motor output (Eq. S2). Thus, when the input map keeps sending spikes to the SC, the saccade continues to grow without bound. To prevent this scenario in the current (linear) model, we tuned down I_0_ together with an increase in input duration (by lowering β). We thus considered two different temporal scenarios:

i. A variable stimulation duration (β) between 0.019 and 0.030 s^−1^ (corresponding to input burst durations between about 300 and 150 ms, respectively), and a current intensity (I_0_) between 0.2 and 3.0 pA, but selected such that the resulting total number of spikes in the SC *output layer* remained invariant (e.g., Fig. 3c,d,g,h (purple curves)).
ii. A similar variation in the input, but now such that the number of spikes sent from the *input layer* to the SC remained constant: β varied between 0.019 and 0.03 s^−1^, and I_0_ between 1.2 and 3.0 pA (e.g., Fig. 3e,f,g,h (green curves)).

Figure 3 illustrates these different external stimulation input scenarios. The first three rows of the left-hand column (Fig. 3a,c,e) show the spatial distributions of the peak firing rates of the neurons in the input population, when the target stimulation point corresponded with T = 15° (i.e., at *u*_*T*_ = 2.5 mm); the right-hand column (Fig. 3b,d,f) shows the temporal profiles of the spiking patterns for the central neuron in the input population at *u*_*T*_ = 2.5 mm. Figure 3a,b shows the spiking responses of the input layer neurons when the external input width was systematically varied between 0.05 and 1.0 mm, and the amplitude between 2.0 and 3.0 pA. The red curve corresponds to the default control stimulation (σ_pop_ = 0.5 mm). To avoid non-physiologically high firing rates, we imposed an upper limit to the evoked firing rates in the input layer (by including a saturating sigmoid input–output relationship) at 400 spikes/s.

Figure 3c,d shows the spike-density functions of the input layer when the input current at *u*_*T*_ = 2.5 mm stimulated a fixed population size (0.5 mm), but with a variable current duration (β) and strength (*I*_*0*_). These latter two parameters were selected such that the SC neural population generated a fixed number of output spikes (Fig. 3h, purple line). Note that the amplitude of the spike density function of the central input neuron decreased with decreasing external current strength; at the same time, burst duration in(de-)creased with de(in-)creasing stimulus strength.

Figure 3e,f shows the input layer responses to the external currents when the input duration and strength were tuned such that the input population sent a fixed number of spikes to the SC. Figure 3g,h illustrates how the number of spikes of the input- (panel g) and output (panel h) layers varies in response to the chosen input currents with variable temporal behavior: a constant number of spikes of the input (green), vs. output layer (purple). Note that if the number of spikes is constant in one layer, it either decreases (input) or increases (output) in the other layer.

### Superior Colliculus cells

The neurons in the SC layer receive the total synaptic input current, given by the synaptical weighted sums of the spikes from the input-layer, and from the SC neurons themselves, whereby the latter are relayed by conductance-based lateral excitatory-inhibitory synapses (equation (S7), in Supporting Information). Because of the location-dependence of the parameters specifying the AdEx equations for the SC neurons, their activity patterns depended on their location in the motor map. Neurons near the rostral site generate a small saccade with a high-frequency, short-lasting burst of activity, while at caudal sites the evoked activity has a lower peak firing rate, and longer burst duration, associated with a large saccade^14^.

#### Lateral intra-collicular connections

The saccade-related neurons in the SC population communicate with each other through lateral interactions, which cause all bursts to approximately synchronize with the central cell^13^. In the original version of the model, these interactions were described by a “Mexican-hat” function (short-range excitation, and long-range inhibition^44^), which acts as a soft winner-take-all mechanism^41^.

Two Gaussians describe the spatial extent of the excitatory and inhibitory connection profiles, between neuron, *n*, and any other neuron, *i*, in the motor map (apart from itself) as function of anatomical position. In the present study, we slightly modified the earlier proposal to:

2a $${w}_{i,n}^{exc}={S}_{n}{\cdot \overline{W}}_{exc}\cdot {\text{exp}}\left(-\frac{{\left|\left|{u}_{i} - {u}_{n}\right|\right|}^{2}}{2{\sigma }_{exc}^{2}}\right) \quad \mathrm{ for } \; n\ne i$$

2b $${w}_{i,n}^{inh} = {S}_{n}\cdot \left(1-{\overline{W}}_{inh} \cdot {\text{exp}}\left(-\frac{{\left|\left|{u}_{i} - {u}_{n}\right|\right|}^{2}}{2{\sigma }_{inh}^{2}}\right)\right) \quad \mathrm{ for } \; n\ne i$$

with ${\overline{W}}_{exc}=0.16 \; \mathrm{nS}$ and ${\overline{W}}_{inh}=1.15 \; \mathrm{nS}$ fixed excitatory and inhibitory weight parameters. The location-dependent gain, *S*_*n*_, causes the lateral interaction scheme to be site-dependent. These lateral connections have a direct effect on the spiking behavior of each neuron, and hence on the overall network dynamics. Strong excitation (re. inhibition) would result in an unbounded spread of the population activity across the motor map (and hence, an ever-increasing saccade amplitude), whereas strong inhibition would quickly fade out the neural activity altogether. We aimed to find parameter values that would ensure a balanced amount of excitation and inhibition, leading to a stable Gaussian population activity, in such a way that (considerable) spatial–temporal changes in the input population activity (as illustrated in Fig. 3) would lead to experimentally observed changes in the SC output saccades (equation (S1)).

### Network tuning

We employed brute-force search algorithms to find suitable values for the lateral inhibitory and excitatory weight parameters, the feedforward projection strengths from input to output layer, and for intrinsic properties of the AdEx equations of the SC neurons.

Besides ${\overline{W}}_{exc}$ and ${\overline{W}}_{inh}$, we also tuned the widths of the Mexican-hat profiles (σ_inh_ and σ_exc_) to yield an appropriate SC population size with synchronized activity, also when the total input activity profile would far exceed the normal default size of 0.5 mm (Fig. 3a). We further extended the model with the lateral synaptic gain parameter, *S*_*n*_, as a location-dependent excitatory and inhibitory scaling.

The intrinsic biophysical parameters of the AdEx equations for the SC neurons (Supporting Information) were optimized by systematically varying their adaptation time constant, τ_q,n_, in combination with the location-dependent feedforward synaptic projection strengths between the layers, ${w}_{n}^{FS}$. In addition, we assessed the effects of varying the location dependence of the intra-collicular scaling parameter, *S*_*n*_, on the saccade trajectories.

The adaptive time constant affects the susceptibility of the neuron to synaptic input and influences its instantaneous firing rate and bursting properties, and hence the kinematics of the saccade. As the feedforward synaptic projection strength between the input layer and SC layer determines the number of presynaptic spikes that is transferred from the input layer to the different locations of the SC layer, it mainly affects the SC neuron’s peak firing rate. The intra-collicular synaptic gain, *S*_*n*_, normalizes the SC output against variability in the total input activity. Together, these three parameters caused a systematic change in the firing properties of SC cells along the rostral-caudal axis of the motor map, while ensuring a fixed maximum number of spikes for the neurons’ preferred saccades, *N*_*u*_*(R)*, with a sigmoid-like response sensitivity to large changes of the input firing patterns.

We employed a similar brute-force search method as employed in^40,41^ to find the optimal location-dependent values of [*τ*_*q,n*_, ${w}_{n}^{FS}$*, S*_*n*_] that ensured a fixed number of spikes per neuron for a saccade that kept a constant amplitude and peak velocity for input patterns far exceeding the default strength of *σ*_*pop*_ = 0.5 mm (e.g., Fig. 3a). Note that the input currents in which we also varied the temporal stimulation properties (β) were not used to tune the parameters of the network.

Equation (3) summarizes the results of the network tuning for the adaptation time constant, *τ*_*q,n*_, and for the top down-projection strengths, ${w}_{n}^{FS}$, as function of the map coordinate, *u*_*n*_. Interestingly, to obtain appropriate saccade responses (see below), both parameters resulted to co-vary in a linear way with the anatomical rostral-caudal location:

3 $${\tau }_{q,n} = 60{-}{12\cdot u}_{n} \; \; \text{and } \;\; {\mathrm{w}}_{n}^{FS} = 10{-}{1.2\cdot u}_{n}$$

**Figure 4:**
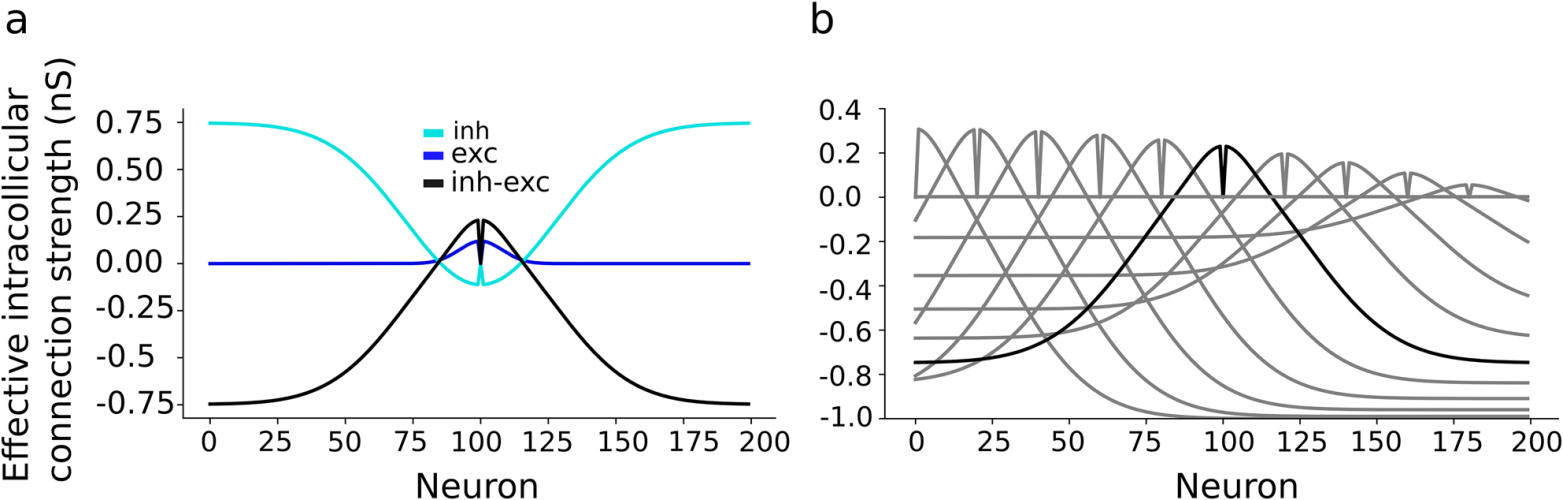
(**a**) The excitatory (σ_exc_ = 0.2 mm; dark-blue) and inhibitory (σ_inh_ = 0.7 mm; light-blue, shown inverted) intra-collicular synaptic connections, and their total effect (black line) for central neuron at neural population generating 15 deg saccade, result in a symmetric local excitatory and global inhibitory connectivity. The net excitation around the neuron (at 0) approaches the value of + 0.23. (**b**) Total effect of excitatory and inhibitory intra-collicular synaptic connections for neurons across motor map generating different saccade amplitude. The intra-collicular synaptic connections are stronger towards the rostral zone (thus counter-acting the higher firing rates) than towards the caudal zone (where cells have lower firing rates).

Figure 4a depicts the net intra-collicular lateral connection strengths from each neuron as obtained from the brute-force search. The lateral connections yield short-range excitatory and long-range inhibitory effects from each neuron in the map. Effectively, SC neurons receive both excitatory and inhibitory potentials from cells endowed with different adaptation time constants, firing rates, and reversal potentials (Supporting Information, S1 Table). Due to the strong symmetric lateral inhibitory connections in the SC layer the number of active neurons in the SC layer saturates when the external input current results in a large recruited input population that may far exceed the standard size of 0.5 mm.

Figure 4b shows the total intra-collicular lateral connection strengths for neurons across rostral to caudal site of the motor map. The lateral inhibitory and excitatory connection strengths decrease from the rostral to the caudal zone by means of the scaling parameter S_n_, which resulted to mainly influence the shape of the nonlinear main-sequence relationship of the model’s saccades between their amplitude and peak eye velocity. The following heuristically obtained relation provided satisfactory results (see “Results”):

4 $${S}_{n} = 1-0.04 {u}_{n}^{2}$$

### Eye-movement trajectories

Eye movements were encoded by the linear ensemble coding scheme of the population activity in the SC motor map (equation (S1)). We applied the one-dimensional efferent motor map of equation (S2) to the new network configuration. The resulting eye-displacement vector, S(t), was smoothed with a Savitzky–Golay filter to compute the instantaneous eye velocity.

## Result

### Bursting behavior of SC AdEx neurons

**Figure 5:**
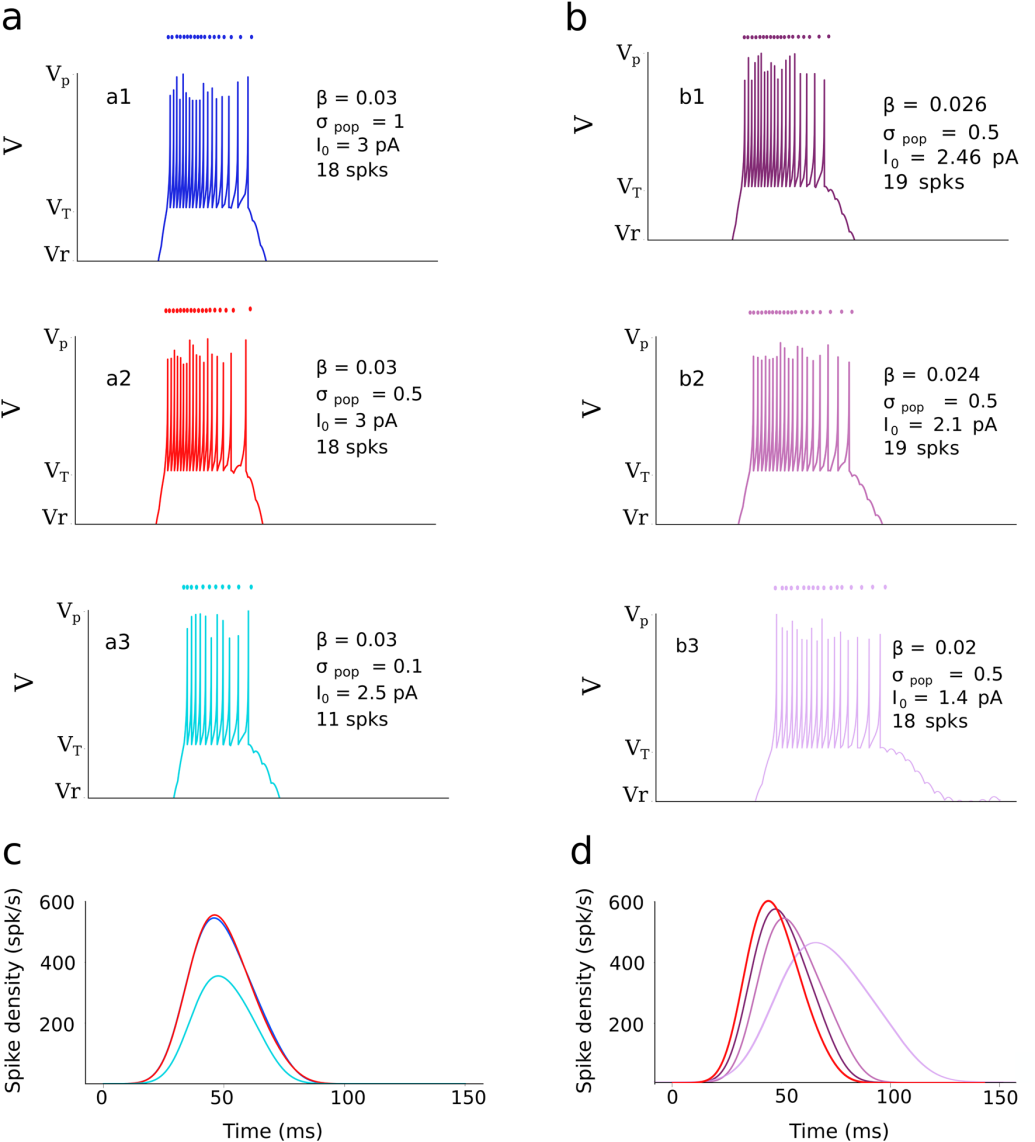
Effect of varying external current input parameters, such as the recruited input population size (σ_pop_), stimulus duration (β), and input stimulus amplitude (I_0_) on the bursting characteristics of an AdEx neuron at u_n_ = 2.5 mm (neuron nr. 100) in the SC output layer [τ_q_ = 30, W ^FS^ = 3]. (**a**,**b**) Show the membrane potential, V(t), and a dot-display of the individual spikes (top), for three input current population sizes around T = 15 deg: (a1) σ_pop_ = 1.0 mm, (a2), σ_pop_ = 0.5 mm (the default stimulation input), (a3) σ_pop_ = 0.1 mm, and for three stimulus duration/intensity values: (b1) β = 0.026/I_0_ = 2.46, (b2) β = 0.024/I_0_ = 2.1, (b3) β = 0.020/I_0_ = 1.4. (**c**) Corresponding spike density functions for varying the external stimulation population size, and (**d**) for varying the external stimulus duration/intensity parameters. Line colors correspond to the traces in (**a**,**b**).

To illustrate the effect of varying the input stimulation (Eq. (1)) on the response behavior of the AdEx model of a typical SC neuron (nr 100), the time dependence of the neuron’s membrane potential, *V(t)*, is shown in Fig. 5, when the input (applied at T = 15 deg) varied in population size, σ_pop_ (Fig. 5a), or in stimulus duration and intensity, β, *I*_*0*_ (Fig. 5b).

The SC neuron in the center of the population (at *u*_*T*_ = 2.5 mm) emitted fewer spikes, *N*_*spk*_ = 11, for the small input population size (σ_pop_ = 0.1 mm; a3), while it generated the same number of spikes, *N*_*spk*_ = 18, for the default input size (σ_pop_ = 0.5 mm; a2) as for the much larger input population (σ_pop_ = 1.0 mm; A1). In all three cases, *V(t)* had the same duration, as the stimulation input current had the fixed default value of β = 0.03 s^−1^. The burst profiles of the neuron (Fig. 4c) for the three different input currents had the same duration too, but the peak firing rate was clearly lower for the small input population.

When the input current was given a fixed population size (0.5 mm) but varied in its duration and intensity parameters (Fig. 5b), the resulting burst durations varied accordingly. The smaller β, the longer the membrane potential, *V(t)*, and, consequently, the resulting burst profile of the neuron. However, in all three cases the emitted number of spikes of the cell remained approximately constant at *N*_*spk*_ = 18 or 19.

### Effect of spatial–temporal changes in the input population

**Figure 6:**
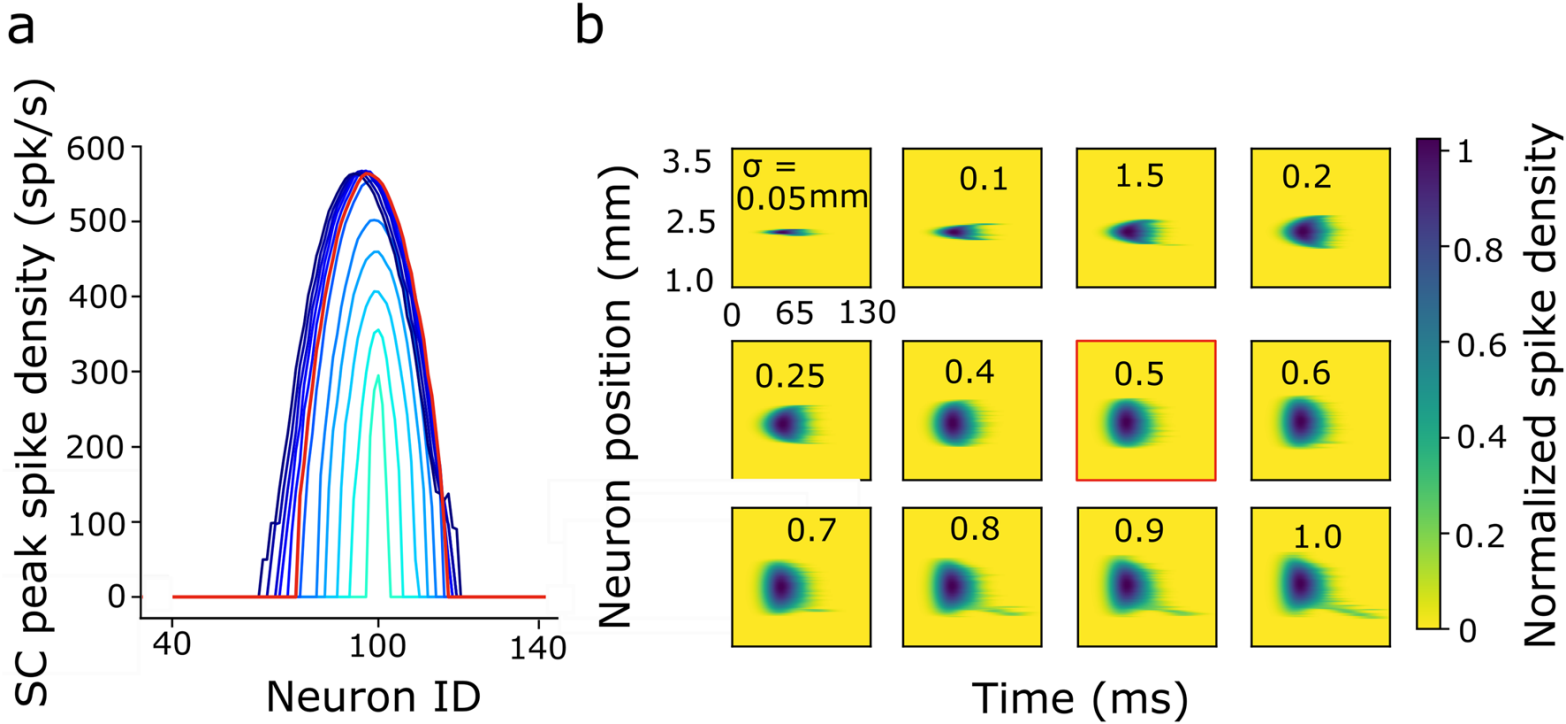
Burst profiles of the neurons in the network with lateral intra-collicular connections in response to the external input current that induces different input population sizes around T = 15 deg. (**a**) Peak firing rates of the SC neural population. Red curve is the response to the default input current with σ_pop_ = 0.5 mm. The population grows with input size up to the default current for low input strengths (light blue), after which it remains approximately invariant (dark blue). (**b**) Firing rate distributions of the neural population in the motor map as function of time, normalized to the absolute firing rate of the central cell (550 spks/s). Each panel shows the result for a specific input population size (indicated in mm). Red outlined panel corresponds to the default stimulation condition (0.5 mm). Panels preceding the default show that the SC population grows with the input population; panels following the default show that the number of active neurons remains approximately constant, even though the input population size grows to the double size.

Figure 6 illustrates the collicular bursting profiles of the cells in the neural population for a saccade towards T = 15 deg for input population sizes, *σ*_*pop*_, ranging from 0.05 to 1.0 mm, with *I*_*0*_ = 2.0–3.0 pA, and β = 0.03 s^−1^ (cf. Fig. 3a). Figure 6a shows the peak firing rates of all recruited neurons in the SC layer for each stimulation condition (color encoded). The red curve corresponds to the default stimulation strength with *σ*_*pop*_ = 0.5 mm and *I*_*0*_ = 3.0 pA. The number of excited neurons, as well as their peak firing rates, increased with increasing input population size, saturating around the default stimulus condition at about 550 spikes/s for the central cell. In Fig. 6b we show the normalized spatial–temporal activity patterns for the entire motor map for each of the different input populations. Note that the burst durations were the same for all stimulus conditions.

The few late spikes that are visible at the rostral end of the population (at the bottom of the panels), especially for the strongest inputs, are due to the rostral-caudal gradient in our revised lateral interaction scheme (Fig. 4; Eq. (4)). This causes slightly more net excitatory input weight to the rostral SC neurons than to the caudal SC neurons. These few extra spikes, however, add very little to the total saccade amplitude and kinematics, which are determined by all spikes within the total population (Eq. S1). In our earlier model ^40^ this late rostral tail was absent as there the lateral interactions were taken identical across the entire motor map (see Fig. 9 in ^40^).

**Figure 7:**
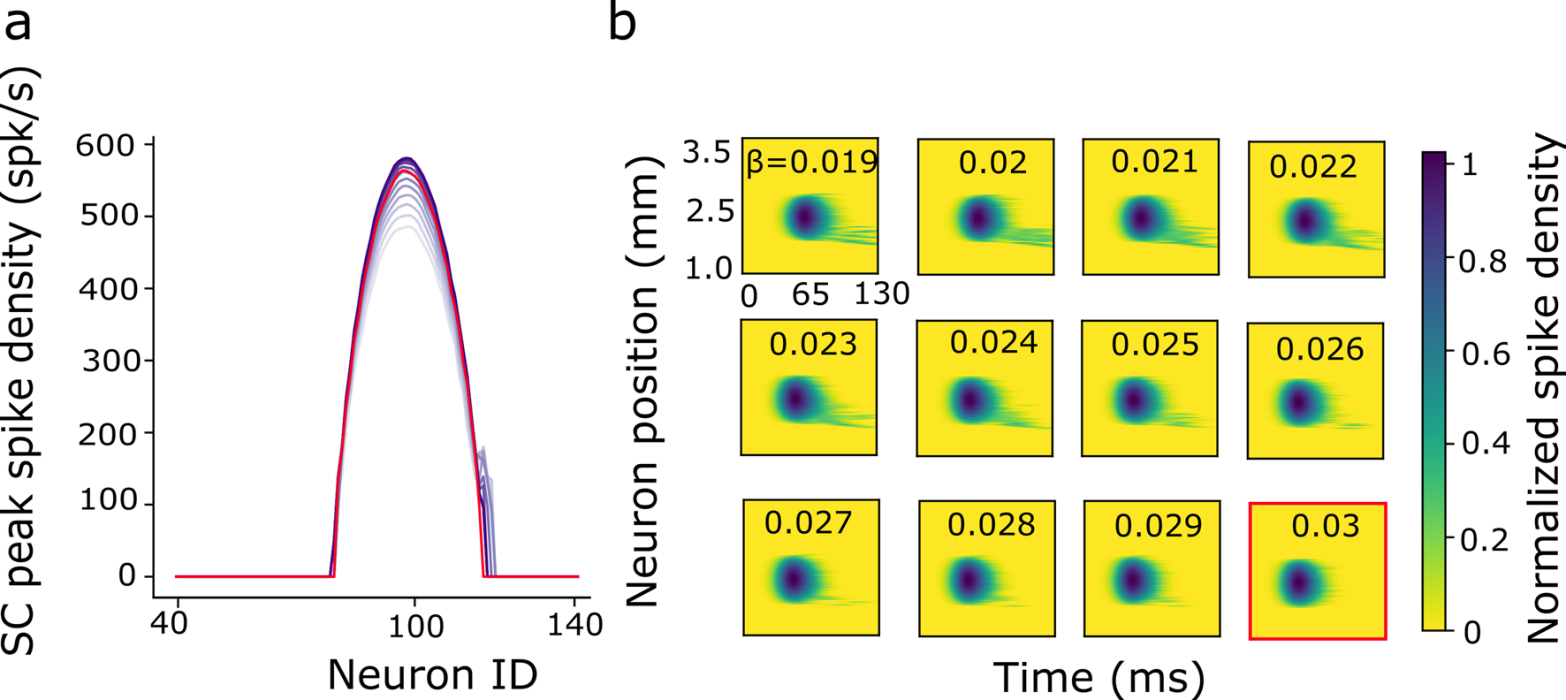
Burst profiles of the SC population in the network in response to the external input current with varying temporal properties (β, I_0_), selected such that the total number of input spikes sent to the SC motor map decreased with increasing β (see Fig. 2c,d). Same format as in Fig. 5. (**a**) Peak firing rates of the neural population in the motor map. Red curve corresponds to the default (β = 0.03 s^−1^). The SC peak firing rate increases with β and reaches a plateau around the default. The total number of SC spikes remained constant (see also Fig. 2g,h, purple data). (**b**) Firing patterns of the neural population as function of time for the different β values. Note that burst durations decrease with increasing β.

Figure 7a,b shows the SC responses across the motor map while varying the duration parameter β of the input stimulus between 0.019 s^−1^ (long) and 0.03 s^−1^ (the default), for a fixed population size (*σ*_*pop*_ = 0.5 mm). The input current intensity, *I*_*0*_, co-varied with β between 1.2 and 3.0 pA in such a way that the number of spikes emitted by the input population decreased with increasing β (cf. Fig. 3c,d, and the purple data points in Fig. 3g,h). As a result, the total number of spikes emitted by the SC population, and hence the saccade amplitude, was independent of β (see also below, and “Methods”, “External input current”).

### Saccade kinematics

**Figure 8:**
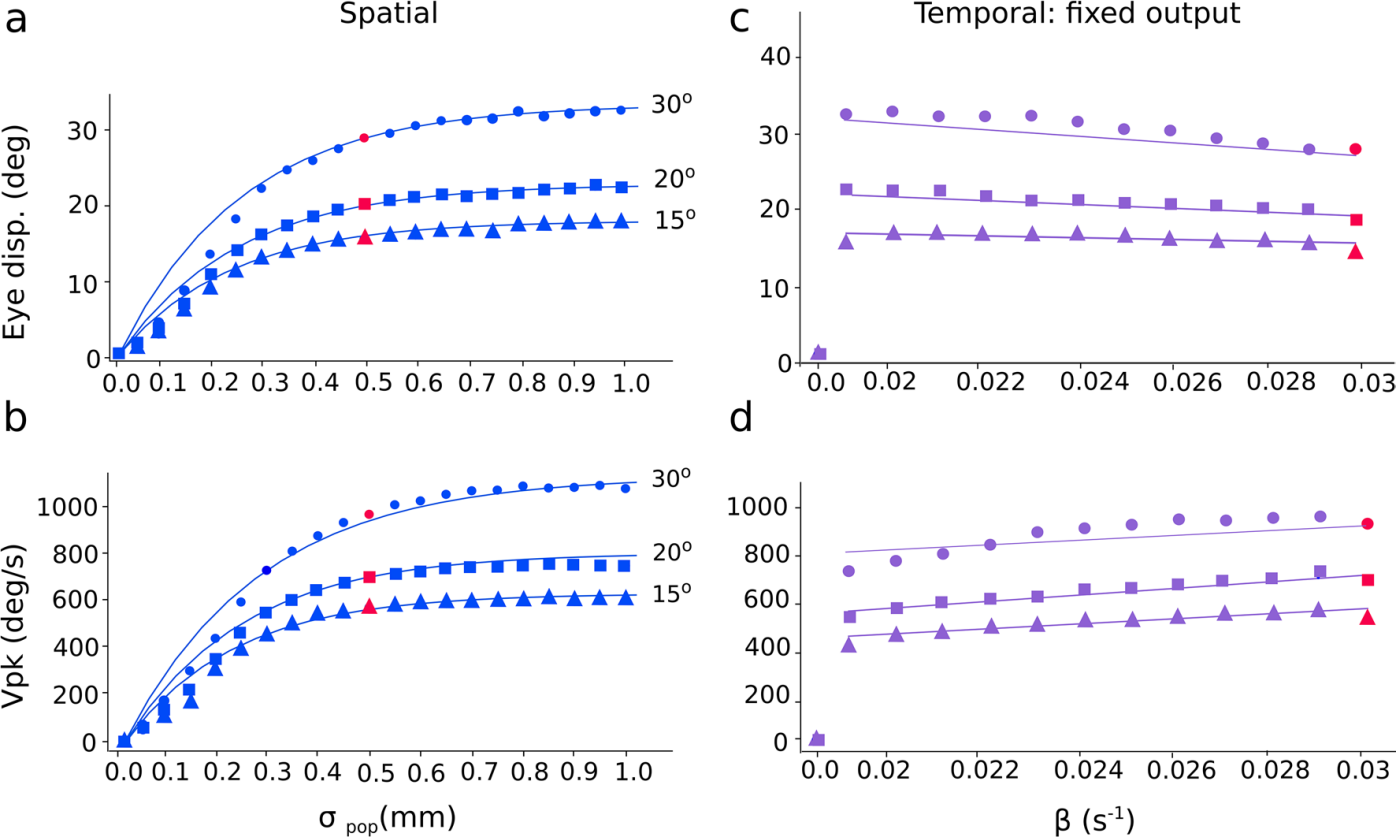
(**a**) Eye-displacement amplitude, and (**b**) peak eye velocity, as a function of the input current’s population size for stimulation at sites corresponding to T = 15, 20 and 30 deg. Beyond the default input population size of σ_pop_ = 0.5 mm (red symbols), the eye displacement amplitude and peak eye velocity are nearly independent of stimulation strength, while below 0.5 mm they both systematically decrease with decreasing input size. (**c**) Eye-displacement amplitude, and (**d**) peak eye velocity, as a function of the input current’s temporal parameter at sites corresponding to T = 15, 20 and 30 deg. The input currents generate fixed number of spikes at SC layer. Whereas the eye displacement remained invariant, peak eye velocity increased with β: the shorter the input duration (large β), the higher the velocity.

In Fig. 8 we show the evoked saccade amplitude and its peak velocity, as a function of the external input current’s population size (Fig. 8a,b), and as function of β (Fig. 8c,d). The input stimulation was applied at three different sites on the input map, corresponding to T = 15, 20 and 30 deg, respectively. When the input population size fell below the default value of *σ*_*pop*_ = 0.5 mm, evoked eye movements fell short of the intended site-specific target location. Around the default size of 0.5 mm (red symbols), the evoked saccade amplitudes approached the final, site-specific values (Fig. 8a). Above the default population size, saccade amplitudes maintained their site-specific size (Fig. 8a) over the full range of input strengths. The associated peak eye-velocity followed a similar input-dependent behavior for changes in the input population size (Fig. 8b). Figure 8c,d show the eye-displacement amplitude and peak eye-velocity as function of β. The input current yielded a fixed population size (0.5 mm) with a variable duration and strength, such as to generate a fixed number of SC output spikes. The evoked saccade amplitudes remained close to the site-specific optimal values for all values of β, which is to be expected when the total number of SC spikes remains invariant. Yet, the peak eye velocity increased slightly with β: short input bursts (i.e., with a high-frequency input stimulation) yielded slightly higher velocities than longer inputs (at low-frequency stimulation).

**Figure 9:**
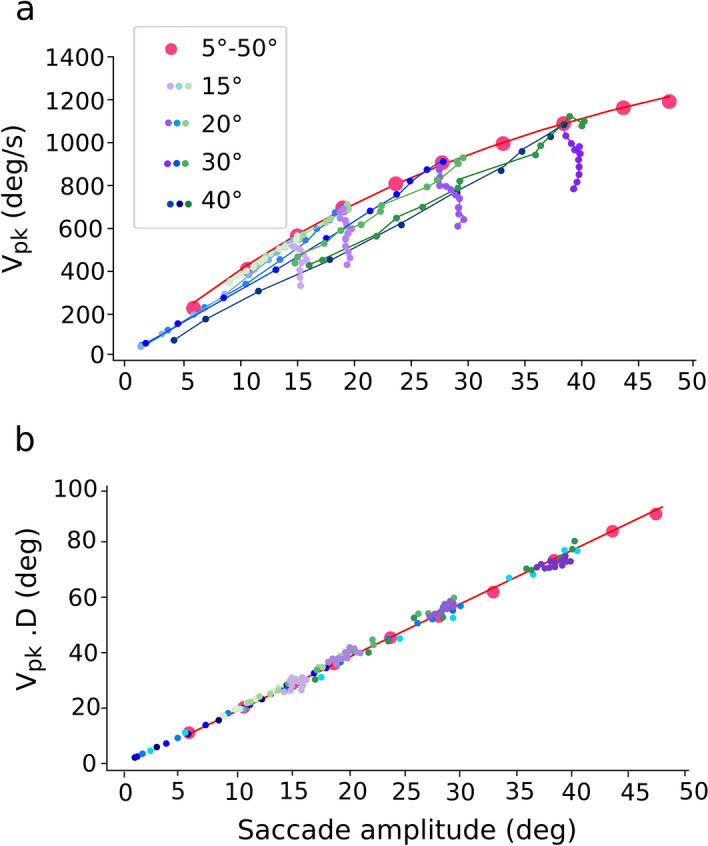
Nonlinear main-sequence behavior of the model. (**a**) Red dots: Saturating amplitude-peak eye velocity relation (Eq. (5)) for the default input current (σ_pop_ = 0.5 mm; β = 0.03; I_0_ = 3.0 pA) applied at 10 different sites. Blue dots: peak eye velocity of saccades evoked at sites T = 15, 20, 30 and 40 deg, respectively, for input currents with different input population sizes (from 0.05 to 1.0 mm), and fixed β. Purple dots: peak eye velocity of saccades evoked at the same sites, for input currents (σ_pop_ = 0.5 mm) with changing (β, I_0_), which kept the number of SC spikes constant. Green dots: same, for input currents with changing (β, I_0_), keeping the number of input-layer spikes constant (compare with Fig. 1a). (**b**) The model saccades all follow the strict linear relationship (Eq. 6) for all stimulation conditions, and for all fast and slow saccades of (**a**).

The kinematic main-sequence behaviors of the model’s saccades are quantified in Fig. 9. The nonlinear amplitude-peak velocity relation of the model is quite comparable to the results from actual saccades, reported for monkey and human^2,36^ (see also Fig. 1a, for a comparison with monkey stimulation data). To quantify the model’s output in response to the default current stimulation applied at different input sites (red dots), we fitted a saturating exponential function:

5 $${V}_{peak}={V}_{0} \left(1-{\text{exp}} \left(-\alpha \cdot R\right) \right)$$

where V_0_ (deg/s) is the saturation velocity for large R, and α (in deg^−1^) is a measure for the slope of the relation near R = 0. The red curve in Fig. 9a corresponds to V_0_ = 1637 deg/s and α = 0.031 deg^−1^. Note that for the different input-stimulation conditions, the evoked saccade amplitudes could vary substantially (see also Fig. 5, for single-cell examples), but the associated peak velocities of these smaller eye movements were also *slower* than for equally sized normal saccades, as all non-default data points fell below the default main-sequence curve (cf. with Fig. 1a). Thus, a fixed site in the SC motor map can generate saccades of different sizes, by variation of the recruited population size. In the model, the latter comes about by weak stimulation of the cells in the input layer. The kinematics of pooled fast and slow saccades have been shown to be well described by the following linear relationship^2,3^:

6 $${V}_{peak}\cdot D=k\cdot R$$

where *k* is the slope (dimensionless) of the relation. In Fig. 9b we applied this relation to the default model saccades (red), obtaining a slope of *k* = 2.0, which is close to the experimentally obtained values for human saccades^2^; it expresses the fact that saccades typically have single-peaked, skewed eye-velocity profiles that resemble a ‘triangular shape’ (cf. Fig. S2d). Figure 9b shows that this relationship also describes all saccade data from the model, as also the smaller and slower eye movements evoked from the different input stimulation parameters all follow the same linear relationship.

## Discussion

### Summary

We studied the properties of a simple, one-dimensional two-layer spiking neural network model with a cortical input and collicular output layer subjected to a large variation in the spiking input patterns. To investigate the relationship between the resulting SC firing patterns, saccade metrics, trajectories, and kinematics as a function of the input current profiles, we varied the input stimulation patterns both in the spatial domain (input population size) and in the temporal domain (input population firing rates and burst durations).

Electrophysiological studies have shown that the saccadic system is quite robust against a large variability in SC input activation, but at near-threshold stimulation levels the evoked saccades become both smaller and slower than expected from the normal main sequence^36,38^. Furthermore, varying the input stimulation frequency, while keeping the total current fixed, modulates the saccade velocity^37^. Our previous spike-count model^40,41^ could not account for these observations, as it was not designed to cope with a large spatial–temporal variation of the input.

By re-tuning the synaptic connectivity between the cortical input and SC output layers, and the intra-collicular excitatory-inhibitory lateral interactions, the new model was able to generate the changes in saccade properties that are associated with the presumed variation in input population size and input firing frequencies, as obtained in electrophysiological studies^36,38^.

### Mechanisms

Once the SC neurons in the model are recruited by the input, local excitatory synaptic transmission among nearby cells rapidly spreads the activation across the motor map to create a neural activity pattern, dictated by the most active central cells in the population. As a result, the burst shapes of the cells within the population were highly correlated^13,41^. Note that the evoked population activity in the SC output layer does not grow without bound, but it is automatically constrained, both in its spatial extent, and in its bursting behavior (peak firing rates), by the inhibitory currents acting on the neurons whenever the external stimulation current attains high values. These inhibitory currents are due to the synaptic far-range lateral inhibition, which ensures that the population size remained within about 0.5 mm in diameter and was largely independent of the applied current when the stimulation parameters exceeded their default values. Conceptually, the lateral interactions normalize the population activity. In our updated network, the inhibitory and excitatory lateral connection strengths decrease (Fig. 4b) from rostral to caudal zone by means of scaling parameter S_n_, thereby influencing the shape (determined by α and V_0_ in Eq. (5)) of the nonlinear relationship between saccade amplitude and peak eye velocity by reducing the firing rates of caudal neurons. It also leads to a small rostral-caudal asymmetry in the population activation, as seen by the low-activity late tail of spikes at the rostral side (Figs. 6b and 7b).

A systematic relationship between input current characteristics and the properties of evoked movements such as amplitude, velocity and duration has been demonstrated in electrical microstimulation experiments in monkey SC^36–38^ (Fig. 1a). These studies reported that the evoked movement amplitude monotonically increased with the stimulation strength for low currents, while saturating at higher current strengths. These input-dependent properties had not been accounted for by our original linear ensemble-coding model^41^, which assumed a fixed Gaussian input pattern, leading to a strong dependence on the input parameters. Yet, the actual electrophysiological results seem to suggest that the external input acts predominantly as a trigger for the SC population-creating process. The intrinsic properties of the SC network subsequently set up the activity patterns of the cells, rather than the details of the external stimulus itself. That is, the effects of electrical stimulation would be mainly caused by intrinsic synaptic transmission, rather than by direct stimulation of the electric field to activate the neurons.

Although a more recent version of the SC model generated an SC population that relied less on the details of the input current, the model could not produce the small-amplitude, slow movements near stimulation threshold. In addition, the input currents were described by stylized rectangular pulses, rather than by realistic spikes from cortical population inputs^40^.

The present spiking-neural network model was able to generate small-amplitude, slower-than-normal saccades at low currents, which increased to a site-specific maximum at higher current strengths. We showed that the intra-collicular lateral connections could be tuned to generate saccades that faithfully followed the nonlinear main-sequence relations of normal, visually evoked saccades (Fig. 8). Importantly, above the default value, the saccade metrics were unaffected by changes in the input stimulation parameters (Fig. 8c). In addition, the saccadic peak eye velocity was also modulated by the temporal properties of the input current: at short input burst durations (i.e., high input burst frequencies, but with a constant number of SC spikes), the evoked saccade velocities were higher, than at longer input (low-frequency) bursts (Fig. 8d).

### Relation single-unit SC activity and ensuing saccade

Note that the linear ensemble-coding model predicts, in its simplest form, that the number of spikes of a given SC neuron for a fixed saccade should always be the same. However, this prediction hinges on the rather strong assumption that a single localized population of SC neurons generates the saccade, of which we can only be sure for a single-target visually evoked saccade in otherwise darkness and no other competing task demands or distractors. In a previous study, we applied this simple idea also to a double-target stimulation task, which can yield a variety of double-step responses, including strongly curved saccades^45^. This work showed that the expected activity for a given SC neuron could vary substantially, even when the overall vectorial displacement of the eye would be identical for all these trajectories. Thus, under such conditions, the strict relationship between SC spiking activity and ensuing saccade metrics and kinematics is broken, even though the saccade could still be generated by the same linear ensemble-coding mechanism.

In contrast, when these highly curved trajectories would result from an intended saccade to a single visual goal, but perturbed, e.g., by a blink response, the SC activity invariably relates to the overall saccade displacement vector, irrespective of the amount of curvature^46^. The reason for this apparent discrepancy in *neural* behavior, despite an overall identical *saccade* behavior, is in the profound differences of the underlying neural program: when the total saccade trajectory results from the sum of two temporally overlapping sub-populations, which are related to the two visual goals, it can be generated in many ways. The problem with such situations is that with a single-unit recording technique it will be impossible to know the potential involvement of other parts of the SC motor map in a double-step scenario, beyond the cells around the recording electrode.

To avoid such ambiguities, we did not consider oculomotor scenario’s that could give rise to several simultaneously activated SC cell populations, other than those leading to a single localized (near-)Gaussian.

Peel and colleagues^35^ observed that after local cooling of the FEF, the total number of spikes of an SC neuron for memory-guided saccades slightly decreased (by about 10% on average) when compared to visual evoked or pre-cooling memory-guided saccades, without affecting the overall saccade metrics. The decrease in spike count was absent for direct visual-evoked saccades. They proposed that the FEF-SC-Brainstem saccade pathway could be (acutely) bypassed by a parallel circuit (possibly involving the fastigial nucleus) to overcome the reduced input to the SC upon local FEF cooling. In this way, the extra signal from the parallel pathway would add to the reduced command from the SC, and still ensure a correct saccade amplitude. As in that case the relationship between the saccade metrics (number of spikes) and kinematics (firing rate) is broken, it may support the idea of a parallel pathway that can compensate for missing SC output. However, use of this alternative pathway is task dependent. Moreover, with the FEF intact, the strict spike-count—saccadic eye-displacement relationship holds for all saccades: slow memory-guided responses and fast direct visual-evoked saccades alike. Hence, under normal conditions, the direct FEF-SC-Brainstem pathway appears to be the major final common circuit for all saccades. This is also in line with the observation that an acute bilateral muscimol-induced inactivation of the SC practically abolishes the monkey’s ability to generate normal saccades^34^.

### Future work

Although our improved model can account for a wide range of saccadic and SC response behaviors under widely different stimulation conditions, it still has several limitations. First, the model should be extended to two dimensions to enable saccades in all directions. The current model architecture allows for a relatively straightforward (but computationally expensive) extension and parameter tuning to a two-dimensional network^40,47^.

A second aspect of real neurophysiological SC firing behavior, missing in our model, is the presence of prelude activity and post-saccadic activity for a large subset of cells. Clearly, these pre- and post-spikes do not contribute to the actual execution of the eye movement, as they don’t reach the saccadic burst generator. In our model, all spikes contribute to the saccade (Eq. S1), and therefore there is no distinction between prelude, burst, and post-saccadic spikes; there is just a single burst in all cells. Thus, to incorporate that SC neurons may also fire before and after the saccade at a relatively low rate will require the presence of separate onset and offset mechanisms that act downstream from the motor map. The former triggers the burst generator (by inactivating the omnipause gate) as soon as the total prelude activity exceeds a certain threshold, whereas the latter will stop the burst generator (i.e., reactivates the omnipause gate) as soon as the subsequent number of SC spikes reaches a fixed level. In^14^ we had shown that the excess of recorded post-saccadic spikes observed in a number of SC neurons would allow for more flexibility of the dynamic spike-counting model to maintain saccade accuracy, e.g., in case of (temporary) local inactivation of the SC.

Possibly, such a potential ‘reservoir’ of extra spikes in the population may also better deal with the considerable noisy variations in the firing behavior of real neurons within the population. Indeed, an important factor that is lacking in the current model is the presence of intrinsic multiplicative and additive noise in the parameters and neuronal dynamics, which would introduce variability in the evoked SC responses and the resulting saccades.

Recently, evidence was provided that in the head-unrestrained monkey the initial eye-in-head position strongly influences the gaze-shift kinematics, and that it has a systematic modulatory effect (‘gain field’) on the SC burst characteristics^48^. Interestingly, the large variation in gaze kinematics for a given gaze-displacement vector, was associated with a similar variation of the SC firing rates: slow gaze shifts were endowed with lower firing rates than fast gaze shifts. Yet, the instantaneous cumulative spike count of the SC cells faithfully encoded the straight gaze (i.e., eye in space) trajectory by following a similar linear relationship as was found for the head-restrained monkey’s eye movements^14^. A more complete model of the SC motor map in gaze control will have to include the control of eye-head gaze shifts, where the contribution of the eye- and head movement will systematically depend on the initial eye-in-head position, and in which an eye position signal modulates the spiking activity within the SC motor map.

## Supplementary Information

Supplementary Information.
