# Supplementary material for: A spiking neural network model of the Superior Colliculus that is robust to changes in the spatial–temporal input
Source: Sci Rep. 2022 Apr 28;12:6916. doi: 10.1038/s41598-022-10991-6 (PMC9050704; doi:10.1038/s41598-022-10991-6)
Supplement: Supplementary file 1 — Supplementary Information. [file 41598_2022_10991_MOESM1_ESM.pdf]

Supporting Information

for:

A spiking neural network model of the Superior Colliculus that is robust to changes in the spatial-temporal input

Arezoo Alizadeh and A. John Van Opstal*

Dept. Biophysics, Donders Centre for Neuroscience, Radboud University
Heyendaalseweg 135, 6525 EZ Nijmegen, The Netherlands

In this Supplemental Material section, we provide the details of the model equations and parameter settings of the neurons in the input layer and SC motor map. We also illustrate some simulation results for stimulation of the model with default input strengths. More detailed background information can be obtained from our earlier work [1].

The linear dynamic ensemble-coding model

The saccade trajectory is generated by dynamic weighting of each spike of each recruited neuron with its efferent connection strength ('spike vector') to the downstream saccade brainstem circuitry, and linearly adding the effects from the total population:

$$S(t) = \sum_{n=1}^{N_{pop}} \sum_{s=1}^{N_{spk,n}<t} m_n \,.\, \delta\left(t - \tau_{n,s}\right) \qquad \text{(S1)}$$

in which $\delta\left(t - \tau_{n,s}\right)$ is a spike of neuron *n* at time $\tau_{n,s}$, N_{pop} is the total number of active neurons in the population, $N_{spk,n}$ is the total number of spikes fired by neuron *n*, and m_n is the cell's site-specific, fixed 'spike vector'. The latter is determined by each neuron's efferent synaptic connection strength to the horizontal brainstem circuitry [2] , which we here simply specified in one dimension as:

$$m_n = \kappa\ A \left[\ \exp\left(\frac{u_n}{B_u}\right) - 1\ \right] \qquad \text{(S2)}$$

with $u_n \in [0 - 5]$ mm the cell's anatomical rostral-caudal location in the SC map, encoding the horizontal saccade amplitude, and κ a fixed scaling factor that only depends on the assumed cell density (in number neurons/mm). This scaling factor was calibrated for a horizontal saccade of 15

deg. The SC afferent mapping parameters (here: A=3.0 deg, and B_u=1.4 mm) had been estimated from [2,3].

We simulated eye movements in response to the presentation of a single input stimulus at a particular location in the input layer. A target point in visual space at T deg from the fovea was thus mapped to its anatomical position, u_T, corresponding to the center of a Gaussian-shaped population in the input layer according to the afferent mapping function of Ottes et al. ([2]; Fig 1, top):

$$u_T = B_u \ln\left(\frac{T+A}{A}\right) \text{ mm} \tag{S3}$$

The AdEx neuron model

The neurons in the network were implemented in the Brian2 spiking neural network simulator [4] with a time resolution of 0.01 ms. We used adaptive exponential integrate-and-fire (AdEx) neurons, which enables realistic bursting dynamics. The AdEx neuron is a simplification of the biophysical Hodgkin–Huxley model, as it contains only two state variables: the membrane potential, V(t), and the adaptation current, q. The dynamics of the AdEx neuron model are determined by two differential equations in which the parameters have a clear physiological interpretation:

$$C\frac{dV_n}{dt} = -g_L(V_n - E_L) + g_L\eta \, exp\left(\frac{V_n - V_T}{\eta}\right) - q_n + I_{syn,n}(t) \tag{S4}$$

$$\tau_{q,n}\frac{dq_n}{dt} = a(V_n - E_L) - q_n \tag{S5}$$

Here, C is the membrane capacitance, g_L is the leak conductance, E_L is the resting potential, η is a slope factor, $I_{syn,n}$ is the total synaptic input current, τ_q is the adaptation time constant, a is the subthreshold adaptation constant, V_T the threshold potential.

In the SC model neurons, two biophysical parameters of the model depended on the location of the neuron (u_n) in the motor map, and thus determined the bursting properties of these neurons: the adaptation time constant, $\tau_{q,n}$, and the synaptic input current, $I_{syn,n}$. The other parameters, C, g_L, E_L, η, V_T and a, were taken location-independent and were optimized such that they ensured an appropriate neural bursting behavior (see Table S1, for the values of all parameters). The AdEx neurons in the input layer all had identical biophysical properties, independent of their location in the input map. Therefore, all SC neuron received similar spiking activity from the input layer across different intended saccades.

Optimal parameter values for the AdEx model neurons, and for all network connections, were obtained from a brute-force algorithm, as there exists no analytical solution for the system.

A spike is triggered whenever the membrane potential, V(t), rapidly grows to infinity. In practice, however, we set a spiking threshold, V_T. The slope factor determines the sharpness of the threshold and implements a smooth spike initiation zone instead of a strict all-or-nothing spiking threshold. For each spiking event at time, τ, the integration of the equation is reset to its resting potential, V_r, and the adaptation current, q, is increased by b to implement the spike-triggered adaptation:

$$V(\tau) \rightarrow V_r \quad , \quad q(\tau) \rightarrow q(\tau) + b \tag{S6}$$

Table S1 Overview of all parameters used in the network simulations

**Input current**		
σ_p	0.05-1 mm	Recruited input population size
β	0.03 s^{-1}	Measure for burst duration
γ	1.8	Skewness of input current profile
I_0	2.4-3 pA	Input stimulation strength
**Input neuron parameters**		
C	50 pF	Membrane capacitance
g_L	2 nS	Leak conductance
E_L	-70 mV	Leak reversal potential
V_T	-50 mV	Spike initiation threshold
Vpeak	-30 mV	Practical spiking threshold
η	2 mV	Spike slope factor
a	0 nS	Subthreshold adaptation
b	60 pA	Spike-triggered adaptation
V_r	-55 mV	Resting potential
τ_q	30 ms	Adaptation time constant
**SC neuron parameters**		
C	280 pF	Membrane capacitance

g_L	10 nS	Leak conductance
E_L	-70 mV	Leak reversal potential
V_T	-50 mV	Spike initiation threshold
Vpeak	-30 mV	Practical spiking threshold
η	2 mV	Spike slope factor
a	4 nS	Subthreshold adaptation
b	80 pA	Spike-triggered adaptation
V_r	-45 mV	Resting potential
τ_q	1-60 ms	Adaptation time constant
**SC synapse parameters**		
E_e	0 mV	Excitatory reversal potential
E_i	-80 mV	Inhibitory reversal potential
τ_e	5 ms	Excitatory conductance decay
τ_i	10 ms	Inhibition conductance decay
w_n^{FS}	4-10 nS	Feedforward synaptic strengths
**Mexican hat parameters**		
W	0.16 nS	Excitatory scaling factor
W [illegible]	1.15 nS	Inhibitory scaling factor
σ_{exc}	0.2 mm	Range of excitatory synapses
σ_{inh}	0.7 mm	Range of inhibitory synapses

Synaptic function of the SC neural model

A SC neuron in the motor map receives total input from spiking activity of its surrounding collicular neurons through conductance-based synapses from the excitatory and inhibitory synaptic transmission, and from spiking activity of the input layer neurons, which in turn are stimulated by an external input current. The excitatory-inhibitory synaptic input to an SC neuron is given by:

$$I_{syn,n}(t) = g_n^{exc}(t)\big(E_e - V_n(t)\big) + g_n^{inh}(t)\big(E_i - V_n(t)\big) \quad \text{(S7)}$$

where g_{exc} and g_{inh} are excitatory and inhibitory synaptic conductances of neuron n; E_e and E_i are excitatory and inhibitory reversal potentials, respectively (for their values, see Table S1).

The excitatory conductances respond dynamically to the activity of the collicular neurons and the external cortical input. They increase for each presynaptic spike by the excitatory synaptic connection strength with nearby collicular neurons, and for each spike from the cortical input neuron at the same location, and afterwards they decay exponentially, according to:

$$\tau_{exc}\frac{dg_n^{exc}}{dt} = -g_n^{exc} + \tau_{exc}w_n^{FS}\sum_{s}^{N_{spk}^{Input}}\delta\left(t-\tau_{n,s}\right) + \tau_{exc}\sum_{i}^{N_{pop}^{SC}}w_{i,n}^{exc}\sum_{s}^{N_{pop}^{SC_i}}\delta\left(t-\tau_{i,s}\right) \qquad \text{(S8)}$$

Similarly, the inhibitory conductance increases with each presynaptic spike from SC neurons with an inhibitory connection strength to neuron n, after which it decays exponentially:

$$\tau_{inh}\frac{dg_n^{inh}}{dt} = -g_n^{inh} + \tau_{inh}\sum_{i}^{N_{pop}^{SC}}w_{i,n}^{exc}\sum_{s}^{N_{spk}^{SC_i}}\delta\left(t-\tau_{i,s}\right) \qquad \text{(S9)}$$

In Eqns. S8 and S9, τ_{exc} and τ_{inh} are the excitatory and inhibitory time constants, respectively; w_n^{FS} is the synaptic strength between input layer neuron *n* and SC neuron *n*; $w_{i,n}^{inh}$ and $w_{i,n}^{exc}$ are the intra-collicular excitatory and inhibitory lateral connection strengths between neurons *i* and *n*, while $\tau_{i,s}$ is the timing of the presynaptic spike in the spike train *s*, $\delta\left(t-\tau_{i,s}\right)$, projecting from collicular neuron *i* to neuron *n*.

The effect of lateral interactions

To establish the functional role of the intra-collicular lateral connections on network performance in response to large changes in the SC input patterns, we here also illustrate the network's behavior without these lateral interactions. Fig S1a shows how the number of active neurons in the SC layer varies with the input population size, when stimulation is applied at target location T=15 deg at different stimulation strengths, corresponding to different population widths (dark-green curve). The number of activated SC cells increases with the input population size up to a level where all 200 SC cells become recruited. Similarly, the total number of spikes emitted by the SC population appears to increase approximately linearly with the population size (light green). This means that the resulting saccade amplitude (Eq. S1), which was tuned to 15 deg for the default population size of 0.5 mm, increases to 27 deg for the input population size of 1.0 mm. Only the maximum number of spikes emitted by the central cell remained constant, because the input firing rates had been limited in the simulations to 400 spikes/s, and the central neuron receives its input spiking activity only from the neuron in the input layer directly above it, through the one-to-one synaptic connections between the two layers (see also Methods, and Fig 1).

Fig S1b shows the results of the network model for the same input stimulation conditions, but now with the lateral interactions as described by Eqns. 2-4. For small input populations (up to 0.3-05 mm), the number of recruited cells, the total number of spikes, as well as the number of spikes of the central neuron, all gradually increase with the input population size. However, the number of activated neurons and the total number of spikes from the motor map increase very little beyond the default stimulation input of 0.5 mm. As a result, the saccade amplitude now follows a saturating relationship with the input stimulation intensity (see also below).

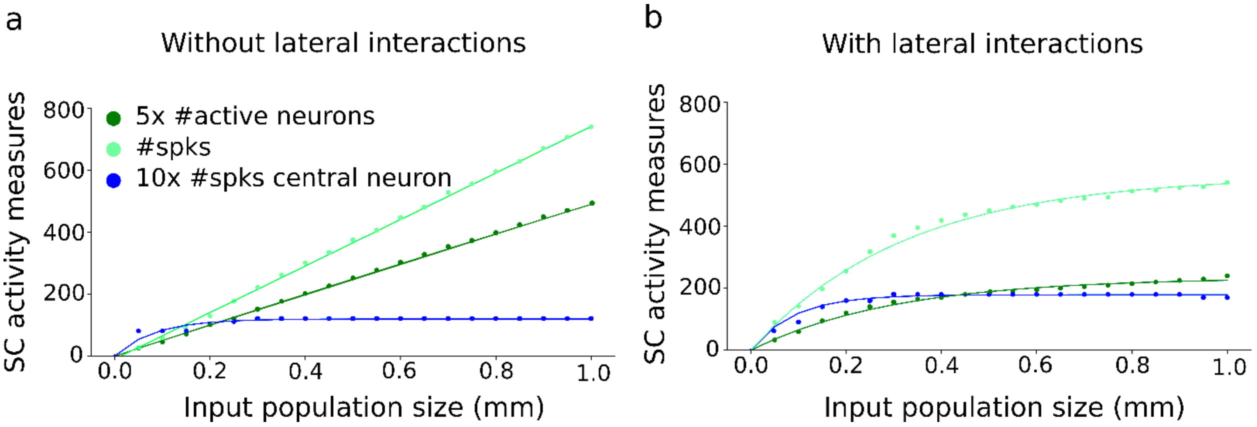

Fig S1. *Number of active neurons (dark green) in the SC layer, the total number of spikes emitted by the neural population (light green), and the number of spikes emitted by the central cell in the population (dark blue) for a saccade towards T=15 deg, as function of the input population size: (a) in the absence of lateral connections, and (b) with the lateral connections specified by Eqns. 2-4. Without lateral connections, the total number of spikes and the number of activated SC neurons increase without bound (R=27 deg,* V_{pk}*=1000 deg/s; not shown). Due to limiting stimulation strength to 400 spikes/s and considering one-to-one synaptic connections between two layers, number of central cell spikes does not increase by increasing external input current's population size. With lateral interactions, the number of spikes from the population, and the recruited population size both saturate (R= 16.2 deg,* V_{peak}*= 580 deg/s).*

Model output for the default input strength

In Fig S2 we quantified the collicular bursts in response to the external input current with default parameters: σ_{pop} = 0.5 mm, I_0 = 3.0 pA, and β = 0.03 s^{-1}, when applied at different sites in the input map. Appropriate tuning of the biophysical parameters of the SC cells, such as $\tau_{q,n}$ and w_n^{FS} along the rostral-caudal axis in the SC map, ensured burst profiles that faithfully reflect the experimentally observed spatial variations in the saccade-related SC bursts [5]. Fig S2a shows how the evoked collicular bursts of the central cells in the recruited population systematically reduce their peak firing rates, and increase their duration, with increasing saccade amplitude. Panel S2b quantifies these physiological properties as function of target eccentricity. According to the linear ensemble-coding model (Eqn. S1), these burst properties underlie the nonlinear kinematic main-sequence properties of the associated saccadic eye movements. The resulting saccades and their

main-sequence kinematic properties are illustrated in panels S2 c,d for eight stimulation sites in the motor map. Note that the saccade duration increased with the saccade amplitude, and that the peak eye velocity increases in a nonlinear way with saccade size. A further characteristic property of the saccades concerns the skewness of their velocity profiles. Typically, the time-to-peak velocity of saccades changes little across a wide range of saccade amplitudes, while the saccade duration increases systematically with the saccade amplitude.

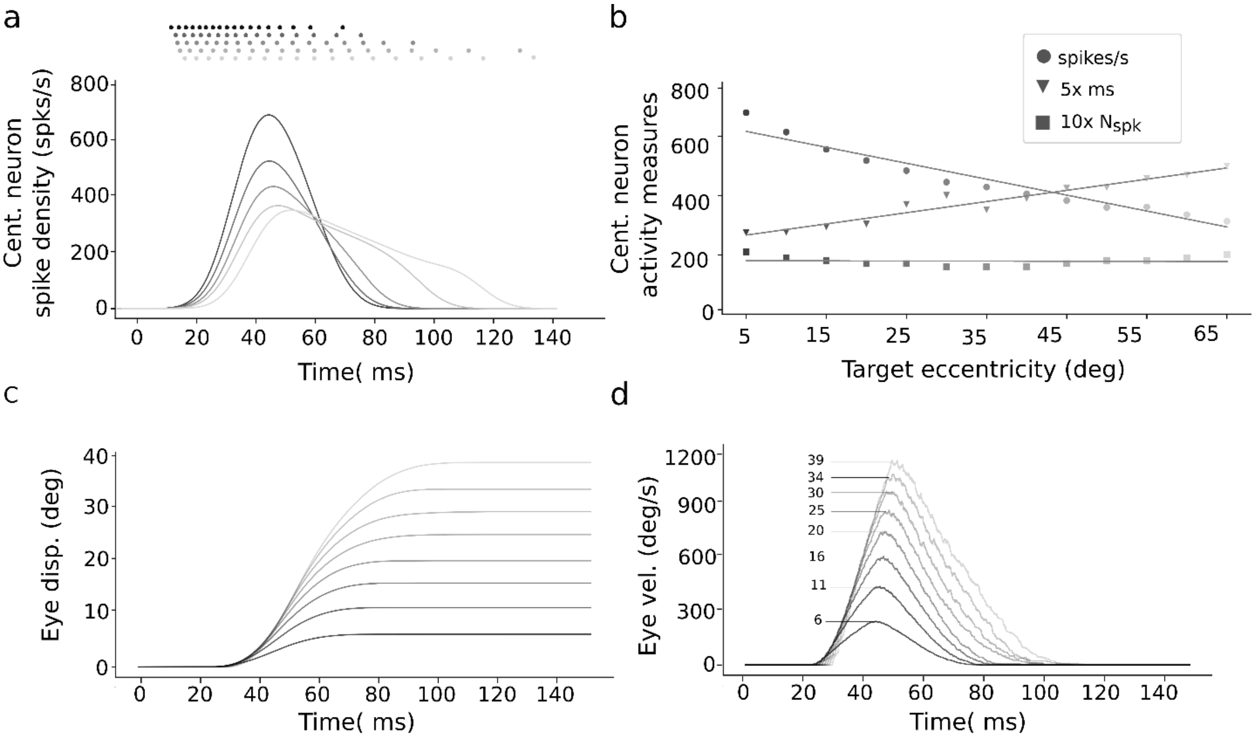

Fig S2. *Results for the default input. (a) Spike trains (top) and spike-density bursts profiles for five different central cells of the SC populations, in response to stimulation with the default input current at 5 different input sites (T=5,20,35,50,60 deg). Note the clear decrease of the peak firing rate, and increase in burst duration, with target eccentricity. The number of spikes in the burst varied slightly, but not systematically, between 16-19 spikes. (b) Central cell peak firing rates (dots) in spikes/s, burst duration (triangles), and the number of spikes (squares) for 13 default inputs applied between T = 5 and 65 deg. Note that the number of spikes for the central cell remains constant throughout the motor map, while the peak firing rate at the caudal sites drops to barely 50% of the rostral stimulation site. The burst durations of the central cell increase monotonically with the evoked movement amplitude. (c) Eye-displacement traces for different targets, at T = 5,10, 15, … ,40 deg, in the input layer. (d) Eye-velocity profiles for the corresponding eye-position traces in (c) (saccade amplitudes indicated). Note the clear increase in saccade duration, and the associated saturation of peak eye velocity as function of saccade amplitude.*

References:

1. Kasap, B. & Van Opstal, A.J. A spiking neural network model of the midbrain superior colliculus that generates saccadic motor commands. Biol Cybern.**111**, 249-268 (2017). https://doi.org/10.1007/s00422-017-0719-9

2. Ottes, F.P., Van Gisbergen, J.A. & Eggermont, J.J. Visuomotor fields of the superior colliculus: a quantitative model. Vision Res. **26**, 857-873 (1986). https://doi.org/10.1016/0042-6989(86)90144-6

3. Robinson, D.A. Eye movements evoked by collicular stimulation in the alert monkey. Vision Res. **12**, 1795-1808 (1972). https://doi.org/10.1016/0042-6989(72)90070-3

4. Goodman, D.F. & Brette, R. Brian: a simulator for spiking neural networks in python. Front Neuroinform. **2**, 5, (2008). https://doi.org/10.3389/neuro.11.005.2008

5. Goossens, H.H. & Van Opstal, A.J. Optimal control of saccades by spatial-temporal activity patterns in the monkey superior colliculus. PLoS Comput Biol. **8**, e1002508 (2012). https://doi.org/10.1371/journal.pcbi.1002508
